# [Retracted] Effect of interventional embolotherapy on FHIT and p16 expression in hepatocellular carcinoma patients

**DOI:** 10.3892/ol.2025.15318

**Published:** 2025-10-06

**Authors:** Wei Xu, Xiaogai Qi, Xia Wang, Jize Sun

Oncol Lett 17: 871–876, 2019; DOI: 10.3892/ol.2018.9648

Following the publication of this paper, it was drawn to the Editor's attention by a concerned reader that the control western blot data shown in Fig. 3 on p. 874 were strikingly similar to data that were already under consideration for publication in another article written by different authors at different research institutes. Owing to the fact that the contentious data in the above article had already been submitted for publication prior to its submission to *Oncology Letters*, the Editor has decided that this paper should be retracted from the Journal. The authors were asked for an explanation to account for these concerns, but the Editorial Office did not receive a reply. The Editor apologizes to the readership for any inconvenience caused.

